# Supplementary material for: Reliability, validity, and responsiveness of the Thai version of the Dry Eye-Related Quality-of-Life Score questionnaire
Source: PLoS One. 2022 Jul 19;17(7):e0271228. doi: 10.1371/journal.pone.0271228 (PMC9295941; doi:10.1371/journal.pone.0271228)
Supplement: S1 Table — DEQS-Th, the Thai version of the Dry Eye-Related Quality-of-Life Score; ROC, the receiver operating characteristic. (DOCX) [file pone.0271228.s001.docx]

**S1 Table. Criterion values of the DEQS-Th and coordinates of the ROC curve.**

| Criterion | Sensitivity | 95% CI | Specificity | 95% CI | +LR | -LR | +PV | -PV | Cost |
| --- | --- | --- | --- | --- | --- | --- | --- | --- | --- |
| ≥0 | 100 | 96.4 - 100.0 | 0 | 0.0 - 11.6 | 1 |  | 76.9 |  | 0.231 |
| >0 | 100 | 96.4 - 100.0 | 10 | 2.1 - 26.5 | 1.11 | 0 | 78.7 | 100 | 0.208 |
| >1.67 | 100 | 96.4 - 100.0 | 20 | 7.7 - 38.6 | 1.25 | 0 | 80.6 | 100 | 0.185 |
| >3.33 | 100 | 96.4 - 100.0 | 23.33 | 9.9 - 42.3 | 1.3 | 0 | 81.3 | 100 | 0.177 |
| >6.66 | 99 | 94.6 - 100.0 | 30 | 14.7 - 49.4 | 1.41 | 0.033 | 82.5 | 90 | 0.169 |
| >8.33 | 98 | 93.0 - 99.8 | 36.67 | 19.9 - 56.1 | 1.55 | 0.055 | 83.8 | 84.6 | 0.162 |
| >10 | 98 | 93.0 - 99.8 | 43.33 | 25.5 - 62.6 | 1.73 | 0.046 | 85.2 | 86.7 | 0.146 |
| >11.66 | 97 | 91.5 - 99.4 | 53.33 | 34.3 - 71.7 | 2.08 | 0.056 | 87.4 | 84.2 | 0.131 |
| >13.33 | 95 | 88.7 - 98.4 | 60 | 40.6 - 77.3 | 2.37 | 0.083 | 88.8 | 78.3 | 0.131 |
| >15 | 93 | 86.1 - 97.1 | 60 | 40.6 - 77.3 | 2.33 | 0.12 | 88.6 | 72 | 0.146 |
| >16.66 | 91 | 83.6 - 95.8 | 66.67 | 47.2 - 82.7 | 2.73 | 0.14 | 90.1 | 69 | 0.146 |
| >18.33 | 90 | 82.4 - 95.1 | 76.67 | 57.7 - 90.1 | 3.86 | 0.13 | 92.8 | 69.7 | 0.131 |
| >21.66 | 85 | 76.5 - 91.4 | 80 | 61.4 - 92.3 | 4.25 | 0.19 | 93.4 | 61.5 | 0.162 |
| >23.33 | 84 | 75.3 - 90.6 | 80 | 61.4 - 92.3 | 4.2 | 0.2 | 93.3 | 60 | 0.169 |
| >25 | 82 | 73.1 - 89.0 | 83.33 | 65.3 - 94.4 | 4.92 | 0.22 | 94.3 | 58.1 | 0.177 |
| >26.66 | 79 | 69.7 - 86.5 | 83.33 | 65.3 - 94.4 | 4.74 | 0.25 | 94 | 54.3 | 0.2 |
| >28.33 | 77 | 67.5 - 84.8 | 86.67 | 69.3 - 96.2 | 5.78 | 0.27 | 95.1 | 53.1 | 0.208 |
| >30 | 68 | 57.9 - 77.0 | 86.67 | 69.3 - 96.2 | 5.1 | 0.37 | 94.4 | 44.8 | 0.277 |
| >31.66 | 65 | 54.8 - 74.3 | 86.67 | 69.3 - 96.2 | 4.88 | 0.4 | 94.2 | 42.6 | 0.3 |
| >33.33 | 62 | 51.7 - 71.5 | 90 | 73.5 - 97.9 | 6.2 | 0.42 | 95.4 | 41.5 | 0.315 |
| >35 | 62 | 51.7 - 71.5 | 93.33 | 77.9 - 99.2 | 9.3 | 0.41 | 96.9 | 42.4 | 0.308 |
| >36.66 | 56 | 45.7 - 65.9 | 93.33 | 77.9 - 99.2 | 8.4 | 0.47 | 96.6 | 38.9 | 0.354 |
| >38.33 | 54 | 43.7 - 64.0 | 93.33 | 77.9 - 99.2 | 8.1 | 0.49 | 96.4 | 37.8 | 0.369 |
| >40 | 51 | 40.8 - 61.1 | 93.33 | 77.9 - 99.2 | 7.65 | 0.53 | 96.2 | 36.4 | 0.392 |
| >41.66 | 49 | 38.9 - 59.2 | 93.33 | 77.9 - 99.2 | 7.35 | 0.55 | 96.1 | 35.4 | 0.408 |
| >43.33 | 47 | 36.9 - 57.2 | 93.33 | 77.9 - 99.2 | 7.05 | 0.57 | 95.9 | 34.6 | 0.423 |
| >45 | 42 | 32.2 - 52.3 | 96.67 | 82.8 - 99.9 | 12.6 | 0.6 | 97.7 | 33.3 | 0.454 |
| >46.66 | 38 | 28.5 - 48.3 | 100 | 88.4 - 100.0 |  | 0.62 | 100 | 32.6 | 0.477 |
| >50 | 35 | 25.7 - 45.2 | 100 | 88.4 - 100.0 |  | 0.65 | 100 | 31.6 | 0.5 |
| >51.66 | 32 | 23.0 - 42.1 | 100 | 88.4 - 100.0 |  | 0.68 | 100 | 30.6 | 0.523 |
| >53.33 | 29 | 20.4 - 38.9 | 100 | 88.4 - 100.0 |  | 0.71 | 100 | 29.7 | 0.546 |
| >55 | 27 | 18.6 - 36.8 | 100 | 88.4 - 100.0 |  | 0.73 | 100 | 29.1 | 0.562 |
| >56.66 | 25 | 16.9 - 34.7 | 100 | 88.4 - 100.0 |  | 0.75 | 100 | 28.6 | 0.577 |
| >58.33 | 24 | 16.0 - 33.6 | 100 | 88.4 - 100.0 |  | 0.76 | 100 | 28.3 | 0.585 |
| >60 | 23 | 15.2 - 32.5 | 100 | 88.4 - 100.0 |  | 0.77 | 100 | 28 | 0.592 |
| >61.66 | 20 | 12.7 - 29.2 | 100 | 88.4 - 100.0 |  | 0.8 | 100 | 27.3 | 0.615 |
| >63.33 | 19 | 11.8 - 28.1 | 100 | 88.4 - 100.0 |  | 0.81 | 100 | 27 | 0.623 |
| >65 | 14 | 7.9 - 22.4 | 100 | 88.4 - 100.0 |  | 0.86 | 100 | 25.9 | 0.662 |
| >66.66 | 10 | 4.9 - 17.6 | 100 | 88.4 - 100.0 |  | 0.9 | 100 | 25 | 0.692 |
| >68.33 | 9 | 4.2 - 16.4 | 100 | 88.4 - 100.0 |  | 0.91 | 100 | 24.8 | 0.7 |
| >71.67 | 8 | 3.5 - 15.2 | 100 | 88.4 - 100.0 |  | 0.92 | 100 | 24.6 | 0.708 |
| >75 | 6 | 2.2 - 12.6 | 100 | 88.4 - 100.0 |  | 0.94 | 100 | 24.2 | 0.723 |
| >78.33 | 5 | 1.6 - 11.3 | 100 | 88.4 - 100.0 |  | 0.95 | 100 | 24 | 0.731 |
| >83.33 | 4 | 1.1 - 9.9 | 100 | 88.4 - 100.0 |  | 0.96 | 100 | 23.8 | 0.738 |
| >86.67 | 1 | 0.03 - 5.4 | 100 | 88.4 - 100.0 |  | 0.99 | 100 | 23.3 | 0.762 |
| >95 | 0 | 0.0 - 3.6 | 100 | 88.4 - 100.0 |  | 1 |  | 23.1 | 0.769 |

DEQS-Th: Thai version of the Dry Eye-Related Quality-of-Life Score; ROC: the receiver operating characteristic
