# Supplementary material for: Reliability, validity, and responsiveness of the Thai version of the Dry Eye-Related Quality-of-Life Score questionnaire
Source: PLoS One. 2022 Jul 19;17(7):e0271228. doi: 10.1371/journal.pone.0271228 (PMC9295941; doi:10.1371/journal.pone.0271228)
Supplement: S2 Table — DEQS-Th, the Thai version of the Dry Eye-Related Quality-of-Life Score; ROC, the receiver operating characteristic. (DOCX) [file pone.0271228.s002.docx]

**S2 Table. Criterion values of the Short Form DEQS-Th and coordinates of the ROC curve.**

| Criterion | Sensitivity | 95% CI | Specificity | 95% CI | +LR | -LR | +PV | -PV | Cost |
| --- | --- | --- | --- | --- | --- | --- | --- | --- | --- |
| ≥0 | 100.00 | 96.4 - 100.0 | 0.00 | 0.0 - 11.6 | 1.00 |  | 76.9 |  | 0.231 |
| >0 | 100.00 | 96.4 - 100.0 | 20.00 | 7.7 - 38.6 | 1.25 | 0.00 | 80.6 | 100.0 | 0.185 |
| >1 | 98.00 | 93.0 - 99.8 | 30.00 | 14.7 - 49.4 | 1.40 | 0.067 | 82.4 | 81.8 | 0.177 |
| >2 | 98.00 | 93.0 - 99.8 | 56.67 | 37.4 - 74.5 | 2.26 | 0.035 | 88.3 | 89.5 | 0.115 |
| >3 | 93.00 | 86.1 - 97.1 | 63.33 | 43.9 - 80.1 | 2.54 | 0.11 | 89.4 | 73.1 | 0.138 |
| >4 | 86.00 | 77.6 - 92.1 | 66.67 | 47.2 - 82.7 | 2.58 | 0.21 | 89.6 | 58.8 | 0.185 |
| >5 | 77.00 | 67.5 - 84.8 | 76.67 | 57.7 - 90.1 | 3.30 | 0.30 | 91.7 | 50.0 | 0.231 |
| >6 | 68.00 | 57.9 - 77.0 | 80.00 | 61.4 - 92.3 | 3.40 | 0.40 | 91.9 | 42.9 | 0.292 |
| >7 | 60.00 | 49.7 - 69.7 | 83.33 | 65.3 - 94.4 | 3.60 | 0.48 | 92.3 | 38.5 | 0.346 |
| >8 | 51.00 | 40.8 - 61.1 | 90.00 | 73.5 - 97.9 | 5.10 | 0.54 | 94.4 | 35.5 | 0.400 |
| >9 | 46.00 | 36.0 - 56.3 | 93.33 | 77.9 - 99.2 | 6.90 | 0.58 | 95.8 | 34.1 | 0.431 |
| >10 | 43.00 | 33.1 - 53.3 | 93.33 | 77.9 - 99.2 | 6.45 | 0.61 | 95.6 | 32.9 | 0.454 |
| >11 | 36.00 | 26.6 - 46.2 | 96.67 | 82.8 - 99.9 | 10.80 | 0.66 | 97.3 | 31.2 | 0.500 |
| >12 | 24.00 | 16.0 - 33.6 | 100.00 | 88.4 - 100.0 |  | 0.76 | 100.0 | 28.3 | 0.585 |
| >22 | 0.00 | 0.0 - 3.6 | 100.00 | 88.4 - 100.0 |  | 1.00 |  | 23.1 | 0.769 |

DEQS-Th: Thai version of the Dry Eye-Related Quality-of-Life Score; ROC: the receiver operating characteristic
